# To be or not to be: The second law of thermodynamics and the flow of life and death

**DOI:** 10.15252/embr.202050861

**Published:** 2020-06-04

**Authors:** Gian‐Paolo Dotto

**Affiliations:** ^1^ University of Lausanne Epalinges Switzerland; ^2^ Massachusetts General Hospital Boston MA USA

**Keywords:** Ecology, S&S: History & Philosophy of Science

## Abstract

Hamlet's question is the artist's expression of the meaning of life. The second law of thermodynamics is the physicist's equivalent.

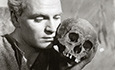

“Habe nun, ach! Philosophie,
Juristerey und Medicin,
Und leider auch Theologie!
Durchaus studirt, mit heißem Bemühn.
Da steh’ ich nun, ich armer Thor!
Und bin so klug als wie zuvor;
Heiße Magister, heiße Doctor gar,
Und ziehe schon an die zehen Jahr,
Herauf, herab und quer und krumm,
Meine Schüler an der Nase herum –
Und sehe, daß wir nichts wissen können!”

I have studied now Philosophy, Law and Medicine
And even, alas, Theology
From end to end, with all my forces.
And now here I am, poor fool,
They call me Magister, they call me Doctor, and it has been at least 10 years
That I have led my students by the nose
And nothing, I see, is given us to know.

Faust's words, at the beginning of Goethe's masterpiece, may ring true to all scientists, physicians, and philosophers and others who have dedicated their life to scientific and other investigations and to education. In all cultures of the past, students of “*Philosophy, Law and Medicine, and even, alas, Theology”* were a privileged class of learned people; they were called to provide knowledge and guidance to others in light of the unpredictability of life and impending disasters, and to heal the afflicted and the sick, and accompany them toward the “after‐life”.

Today, the physician is usually consulted before the priest or the philosopher and can take their place entirely. However, none of them, nor the scientist, can claim to know everything of their profession, let alone the bigger questions about life and the meaning therein. Faust's quest of knowledge and education are undermined by his own admission: “*and now here I am, poor fool….”*. With exponentially increasing knowledge and complexities comes the need to find some unified view to direct our search and actions. Faced with chaos, we look for order; faced with random and haphazard events of nature as well as men, we need to identify some firm ground and some universal laws to explain the world and make sense of unpredictable and complex events.

Faust's quest of knowledge and education are undermined by his own admission: “*and now here I am, poor fool…”*.

## The laws of physics

To help Faust find his way, one could go back to the “laws of nature”, starting from the laws of physics: solid reference points on which everything else depends: chemistry, biochemistry, biology, sociology, and so on. This author, when teaching the basics of biochemistry to hundreds of medical students each year, begins with a simple description of the second law of thermodynamics.

A chemical reaction that generates a product of higher “stocked” energy than the combined energy of the starting molecules cannot happen—or, to state it more precisely, it is very unlikely to happen. A final product of higher energy can only be generated by two coupled reactions, one of which depletes the energy of its substrates in order to increase the energy of the other reaction's product. Based on this postulate, the teacher of biochemistry moves on to explain to students the “quasi‐stationary” equilibrium of cells and living systems. It is based on a dynamic flux of reactions and products, a marvel of spatial and temporal organization of myriads of components, each linked directly or indirectly to the others.

Diseases creep in when this equilibrium is perturbed. There are many external causes that can push such a complex system out of balance, but there are also internal factors that whittle down the robustness of the system until it ultimately fails. Hence, the second law of thermodynamics has general implications for ultimate questions of life and death, on which the teacher and his students have no time to dwell. Those who go on to become physicians will later learn principles of ethics and human care in separate studies. Sooner or later, however, the indissoluble connection between molecular reactions and “higher human needs” will come to the fore. Diseases and failures require small molecules and drugs as much as the physician's personal care and attention.

… the second law of thermodynamics has general implications for ultimate questions of life and death, on which the teacher and his students have no time to dwell.

The second law of thermodynamics was first glimpsed at by the physiologist and physician Herman von Helmholtz, who pondered on the relationship between the heat of animal bodies and their food combustion and the similar operating principles of steam engines. To put it simply, there cannot be a transfer of heat from colder to warmer bodies but only in the opposite direction. Water at room temperature never forms ice cubes, whereas ice cubes added to warm water quickly melt and disappear.

What is the link between heat and energy and life and death? The biochemistry professor will tell the students that it can all be explained by the movement of molecules. Heat can be equated to the kinetic energy of molecules. The greater the heat, the more they move, obeying laws of statistical probability fixed in rigorous mathematical equations. Stored energy, which can generate heat, is increased by restricting the movement of molecules and pressing them against each other. Similar to a compressed spring, this potential energy can be released to do work, just as compressed, heated steam is released to drive a turbine and produce electricity. In this way, accumulated heat and energy sustain the body and feed the mind. Both come to a stop with the cold touch of death.

Are we just the lucky outcome of a random sequence of events, or the result of inevitable natural laws that consume energy to create ever more complexity?

## The progression of life

Any form of life is built on complexes of atoms and molecules and their dynamic association and dissociation. Biologists and physicists invoked the idea of self‐crystallizing networks to explain the origin of life 3.8 billion years ago, 8 billion years after the explosion of energy and its consolidation into time and space: “the Big Bang, the ultimate hero of low frequency” as the Swiss band Yello described it (*Solar Driftwood* in *Pocket Universe*). Hydrodynamic crystals of sulfur–iron compounds in warm water springs would have formed protective cages that harbored chemical reactions which eventually created self‐replicating entities. These cages, with RNA molecules at the center, may have been the origin of cells or the ancestors of viruses before the first cell formed 1.

Even today, the birth of new life is still a mystery. What is the driving force that makes an apparently dead seed wake up under the sun, draw water, and germinate into a plant? Or what is the pulling force that brings a new human being to life? At the end, it is the result of a random process that obeys the second law of thermodynamics and goes beyond. One of our father's sperms, out of billions, fused with one of our mother's eggs and triggered a cascade of events that led to who we are. To quote the title of Jacques Monod's famous book, we are indeed the product of *chance* but also, somehow, of *necessity* 2. While the chance of our coming to be part of life is easy to understand, there is also a necessity that makes us uniquely different and irreplaceable.

In the global scale of time and space, mankind occupies just a tiny spot. Are we just the lucky outcome of a random sequence of events, or the result of inevitable natural laws that consume energy to create ever more complexity? Evolution is not a human process, and endowing it with some kind of intelligent design is an anthropomorphism and plainly wrong. And yet, we are bequeathed with the capacity to probe into the universe and project to its beginning and its end. We can look at and try to understand these evolutionary processes only starting from our own, human perspective as our intelligence is “programmed” to connect and adjust to reality and its challenges. Being immersed in them, are energy, matter, and their interconnection only concepts of the human mind?

In Aristotle's words, *matter* is potentiality that becomes reality by *form*, the two being linked by *efficient* and *final* causes. There is not a marble statue that is only matter, nor is there a marble statue that is only form. The “*final cause*” or end result is the statue itself. The “*efficient cause*” or determining factor is the sculptor who carves the marble. And yet, the sculptor may not have a preconceived idea of what his chisel will produce, but is compelled by some internal compulsion to use it. As Henri Bergson pointed out, without any conventional teleological implications, evolution is driven by an unstoppable energy that pushes life 3. This often‐misunderstood philosopher turned thinking of evolution upside down, when suggesting—perhaps not jokingly—that it is not man's brain at the top of this process, but it is the gonads, filled with germ cells bursting to give rise to new life.

There are baffling similarities and differences between self‐organizing crystals of inorganic matter and life: “reproducing crystals” with a built‐in drive to attract and repel each other and adapt and change in their expansion. Erwin Schrödinger made it clear at the beginning of his influential book *What is life?* that this drive is based on the laws of physics but goes beyond 4. Modern physics is embroiled in a Promethean quest of a theory of everything and assumes that biological systems may be ultimately explained by it. Most biologists likely agree though that human beings are more than equations and probabilistic determinations.

And yet, in the large scale of things, disruptive forms of life are never the final word and other forms of life, sooner or later take over.

## The rule of the second law

Relative to this, the second law of thermodynamics seems something of the past. Its mathematical formulation may not be relevant to our quest for sense and directions. However, the law states that the greater movement in all directions, which can be called disorder (*entropy*), is “in all likelihood” determined to increase at the expense of higher organization, which is order (*enthalpy*). It is a grim view, which postulates that all forms of life will ultimately come to an end. Order and disorder apply to the organization and function of molecules, the human body and mind, but also thought and society. Artistic expression can also be viewed as the result of a continuous struggle between order and disorder; in fact, mathematical calculations of entropy and enthalpy have recently applied to interpret a Jackson Pollock painting 5.

Life, in its unstoppable push forward, brings new forms of order and reasons of hope. However, life can also create disorder and become the source of its own demise. A groomed garden requires a lot of attention and efforts, as rambles can always sprout again. At any age, cancer cells can all of a sudden appear in the human body and, in spite of all attempts to fend them off and restore order, eventually take over and kill the body. Today, we are being painfully reminded that even a tiny virus has the disruptive power to not only kill cells and bodies but to bring the global human society and economy to a halt. And yet, in the large scale of things, disruptive forms of life are never the final word and other forms of life, sooner or later take over. In a deserted Venice in the grip of a viral pandemics, wild ducks roam the streets. The Black Death that ravaged Europe during the 14the century was followed by the Renaissance and the flourishing of the arts and science.

**Figure 1:**
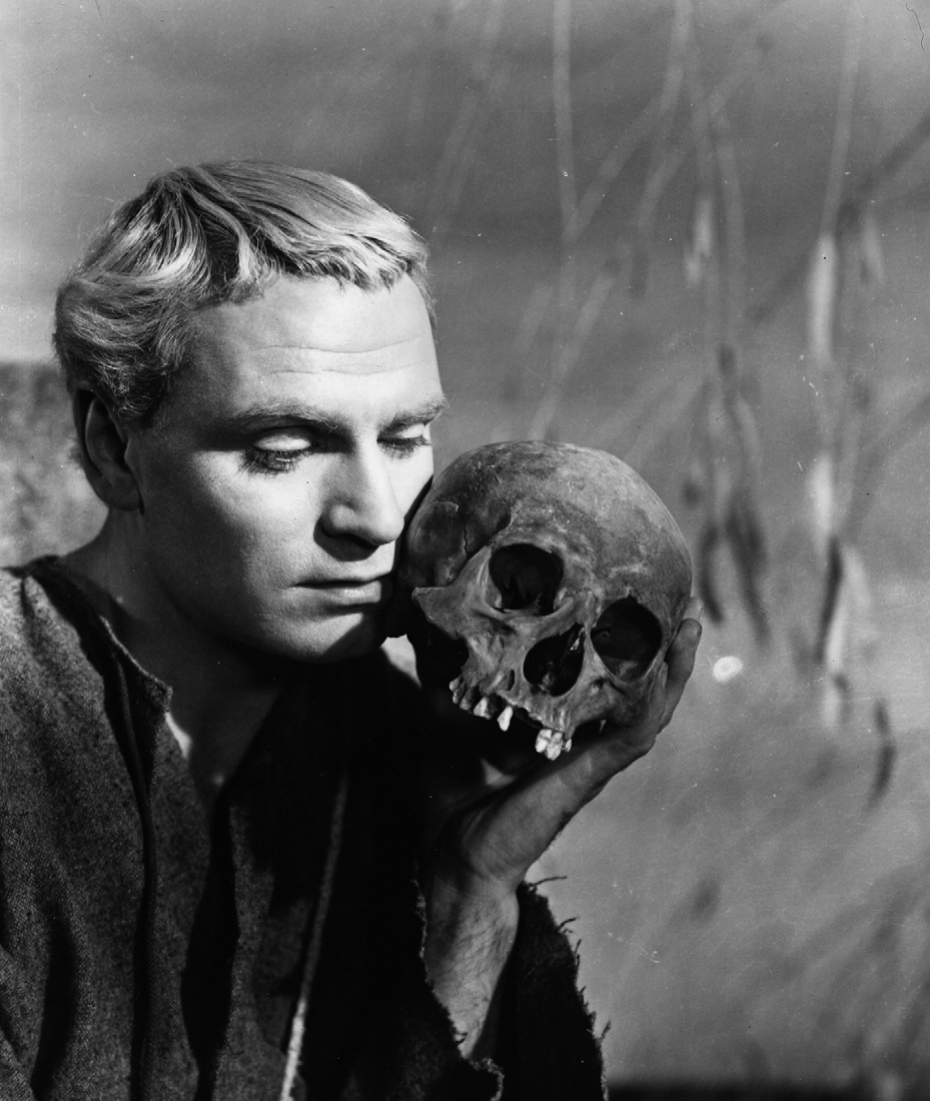
**British actor Laurence Olivier as Hamlet (1948).** © Pictorial Press Ltd/Alamy Stock Photo.

At the end of all thoughts and investigations, we are confronted with a simple question: *To be or not to be*? This is what Hamlet asks himself, holding his father's skull in the hand (Fig 1). This is what we are prompted to ask, in front of life's joys and impending failures. But is this just a fallacious question? *Being* and *not being* may not be two mutually exclusive states but two sides of the same coin. Both, when the coin flips over and we come to life and when it flips the other side and we die, we are still part of the same coin and still remain part of the larger scheme of life and death that drive evolution and progress.

To the inquiring mind, the beginning seems very much like the end: One begets the other. “*Abyssus abyssum invocat*” (abyss calleth unto the abyss): This is a quote from Psalm 42 and the title of a music album by the Polish extreme metal band Behemoth. A bit of silence, rather than rock music, is what is needed though, and perhaps Faust can come back to his senses by pausing for a moment. Seeking and finding the meaning of life may be the equivalent of diving, with confidence, into the ocean. Those who grope and flail desperately end up drowning but those who give themselves in to the water will be carried by it. It is by letting go, that we can avoid the abyss and come back to the surface.
